# LCAT in Cancer Biology: Embracing Epigenetic Regulation, Immune Interactions, and Therapeutic Implications

**DOI:** 10.3390/ijms26041453

**Published:** 2025-02-10

**Authors:** Manzhi Gao, Wentian Zhang, Xinxin Li, Sumin Li, Wenlan Wang, Peijun Han

**Affiliations:** 1 Department of Aerospace Hygiene, School of Aerospace Medicine, Air Force Medical University, Xi’an 710032, China; manzhi1535675200@163.com (M.G.); wentian509@163.com (W.Z.); lxx1628180098@163.com (X.L.); lisumin15229806900@outlook.com (S.L.); 2 Key Laboratory of Aerospace Medicine of Ministry of Education, Air Force Medical University, Xi’an 710032, China

**Keywords:** *LCAT*, HDL, cancer metabolism, immune modulation, epigenetic alterations, personalized cancer therapy

## Abstract

Lecithin cholesterol acyltransferase (LCAT) is a crucial enzyme in high-density lipoprotein (HDL) metabolism that is often dysregulated in cancers, affecting tumor growth and therapy response. We extensively studied *LCAT* expression in various malignancies, linking it to clinical outcomes and genetic/epigenetic alterations. We analyzed *LCAT* expression in multiple cancers and used the Cox regression model to correlate it with patient survival metrics, including overall survival (OS), disease-specific survival (DSS), and progression-free interval (PFI). We also examined the copy number variations (CNVs), single-nucleotide variations (SNVs), DNA methylation, and N6-methyladenosine (m6A) modifications of *LCAT* and their connections to tumor immune responses and drug sensitivity. *LCAT* expression varies among cancers and correlates with patient outcomes. Low expression is linked to poor prognosis in low-grade glioma (LGG) and liver hepatocellular carcinoma (LIHC), while high expression is associated with better outcomes in adrenocortical carcinoma (ACC) and colon adenocarcinoma (COAD). In kidney renal papillary cell carcinoma (KIRP) and uterine corpus endometrial carcinoma (UCEC), *LCAT* CNV and methylation levels are prognostic markers. *LCAT* interacts with m6A modifiers and immune molecules, suggesting a role in immune evasion and as a biomarker for immunotherapy response. *LCAT* expression correlates with chemotherapeutic drug IC50 values, indicating potential for predicting treatment response. In ACC and COAD, *LCAT* may promote tumor growth, while in LGG and LIHC, it may inhibit progression. *LCAT* expression and activity regulation could be a new cancer therapy target. As a key molecule linking lipid metabolism, immune modulation, and tumor progression, the potential of *LCAT* in cancer therapy is significant. Our findings provide new insights into the role of *LCAT* in cancer biology and support the development of personalized treatment strategies.

## 1. Introduction

*LCAT* facilitates the esterification of free cholesterol and its storage in the core region of HDL particles, promoting the maturation and size expansion of HDL particles. This process not only provides substrates for cholesterol reverse transport mediated by cholesterol ester transfer protein but also enhances the functionality of HDL particles. Notably, over 90% of cholesterol esters in plasma are generated through *LCAT* catalysis. In addition to its pivotal role in cholesterol esterification, *LCAT* also hydrolyzes phosphatidylcholine and oxidized platelet-activating factors, thereby effectively safeguarding platelet function and the antioxidant capacity of HDL [1]. Cholesterol, an essential lipid molecule in living organisms, plays a crucial role in maintaining cell membrane integrity, regulating membrane fluidity, and participating in bile acid and steroid hormone synthesis. Recent studies have revealed that tumor cells reprogram cholesterol metabolism pathways to meet the demands of rapid proliferation. This metabolic reprogramming not only directly influences the biological behaviors of tumor cells, such as proliferation, invasion, and metastasis, but also modulates immune cell function by altering cholesterol distribution within the tumor microenvironment, thereby affecting the body’s anti-tumor immune response. These findings provide new insights for developing tumor treatment strategies based on cholesterol metabolism regulation [2,3]. Recent research has begun to shed light on the role of *LCAT* in cancer biology, with findings indicating that *LCAT* activity may be disrupted in a variety of malignant tumors, potentially affecting tumor progression and response to therapy [4,5,6]. However, the exact mechanisms by which *LCAT* contributes to carcinogenesis, as well as its clinical significance across various cancer types, have yet to be fully understood. Because of its function in reverse cholesterol transport and antioxidant action, HDL is frequently referred to as “good cholesterol” [7]. According to new research, HDL malfunction may be linked to a higher risk of cancer and a worse prognosis [8]. An essential enzyme for the maturation and proper operation of HDL is LCAT [9].

Our knowledge of the function of *LCAT* in cancer is severely constrained by the paucity of thorough studies on *LCAT* expression and activity across different tumor types. This study uses systematic approaches to clarify the roles of *LCAT* in tumor growth in light of the diversity of cancer and the intricacy of lipid metabolic pathways. The aim of this study is to investigate *LCAT* expression patterns in different cancer types and their correlation with clinical outcomes, like survival. Additionally, we examined the genetic and epigenetic modifications of *LCAT* in cancer and their impact on the biological properties of tumors.

We examined *LCAT* expression in different cancer types using extensive genomic databases and cutting-edge bioinformatics methods, and we connected the findings to clinical characteristics. Our findings show intricate *LCAT* expression patterns. In some malignancies, prior research has demonstrated a strong correlation between *LCAT* levels and patient prognosis [8,10,11,12,13,14]. We further investigated the genetic underpinnings of *LCAT* dysregulation, including single nucleotide and copy number variations, and their possible effects on the development and spread of tumors. Additionally, we also looked at the potential of *LCAT* as a predictive biomarker of immunotherapy response and its connection to the immune response in the tumor microenvironment.

The goal of this research is to present a thorough analysis of the participation of *LCAT* in cancer while also shedding light on its therapeutic relevance and mechanisms of action. Understanding the tricky relationship between *LCAT*, lipid metabolism, and most cancers’ progression is fundamental for the improvement of centered treatments and might also pave the way for customized therapy techniques that harness the doable of modulating *LCAT* recreation in most cancer treatments.

## 2. Results

### 2.1. LCAT Expression Analysis in Normal and Tumor Tissues

**Figure 1:**
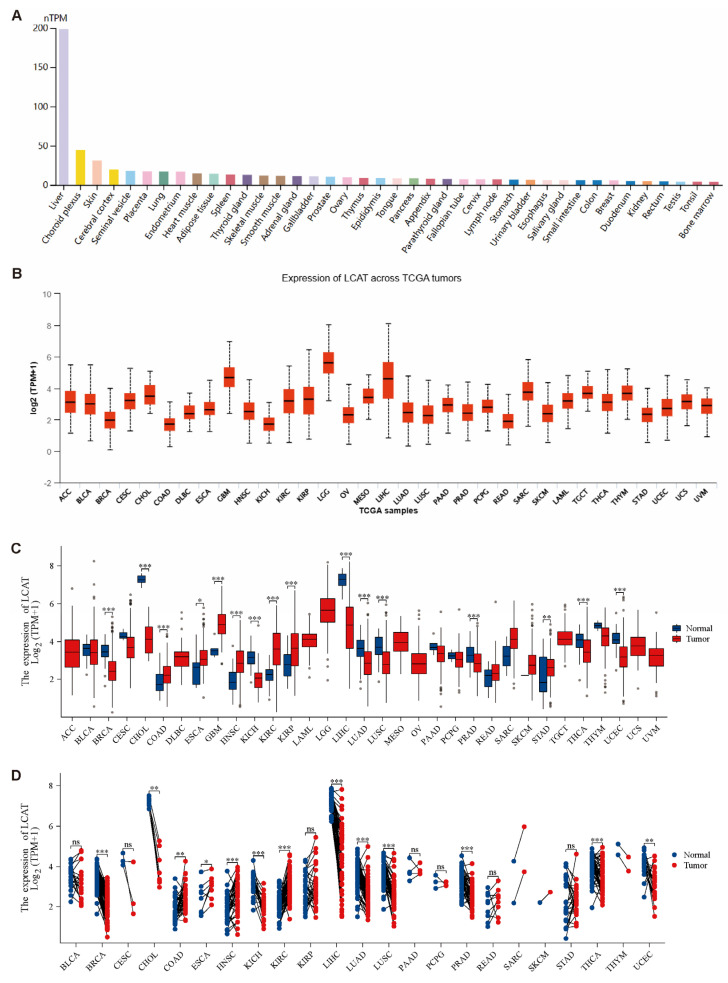
Differential expression analysis of *LCAT* in various tissues. (**A**). Expression of *LCAT* in normal tissues. (**B**). Expression of *LCAT* across TCGA tumors. (**C**). Differential expression of *LCAT* between normal and tumor tissues. (**D**). Differential expression of *LCAT* between tumor tissues and paired adjacent normal tissues. * *p* < 0.05; ** *p* < 0.01; *** *p* < 0.001; ^ns^ *p* < 0.05.

In Figure 1A, our analysis of *LCAT* expression in normal tissues revealed that *LCAT* is most highly expressed in normal liver tissue and least in bone marrow tissue. Further analysis of *LCAT* expression in multiple tumors from the TCGA database found that *LCAT* expression is highest in Brain Lower Grade Glioma tissue (Figure 1B). Compared to normal tissues, *LCAT* expression is reduced in various tumors. Notably, *LCAT* expression is significantly lower in breast invasive carcinoma (BRCA), cholangio carcinoma (CHOL), kidney chromophobe (KICH), LIHC, lung adenocarcinoma (LUAD), lung squamous cell carcinoma (LUSC), prostate adenocarcinoma (PRAD), thyroid carcinoma (THCA), and UCEC tumor tissues; *LCAT* expression is significantly higher in COAD, esophageal carcinoma (ESCA), glioblastoma multiforme (GBM), head and neck squamous cell carcinoma (HNSC), KIRC, KIRP, and stomach adenocarcinoma (STAD) tumor tissues (Figure 1C). Differential expression analysis of *LCAT* between tumor tissues and paired normal tissues yielded similar results (Figure 1D). *LCAT* is lowly expressed in BRCA, LIHC, LUAD, PRAD, THCA, and UCEC tissue samples.

Comparing *LCAT* expression across different pathological stages in 33 tumors revealed that *LCAT* expression in COAD is significantly higher in stage 3 compared to stage 2; *LCAT* expression in LIHC is significantly lower in stages 2, 3, and 4 compared to stage 1 (Figure S1A,B).

**Figure 2:**
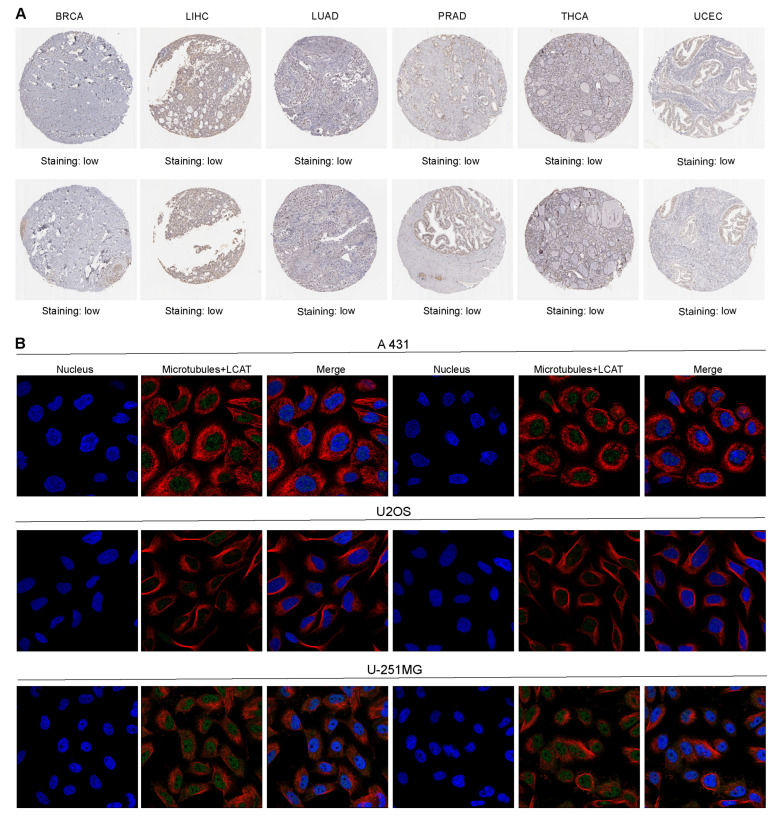
Expression of LCAT in tumor tissue samples and cells. (**A**). Representative immunohistochemical images of LCAT expression in tumor tissue samples (two images for each cancer type). (**B**). Immunofluorescence images of LCAT in cervical cancer cell line (A431), osteosarcoma cell line (U20S), and glioblastoma cell line (U-251MG). LCAT is marked with green fluorescence, microtubules with red fluorescence, and cell nuclei with blue DAPI staining.

As shown in Figure 2A, the immunohistochemical results from the HPA dataset indicate that LCAT is lowly expressed in BRCA, LIHC, LUAD, PRAD, THCA, and UCEC tumors, which is consistent with the findings from the Xiantao Academic website. Immunofluorescence experiments showed that LCAT is located in the nuclei of cervical cancer cell line A431 and glioblastoma cell line U-251 MG and is almost not expressed in malignant bone tumor cell line U20S (Figure 2B). Protein localization data generated from the Human Protein Atlas also indicate that LCAT is primarily localized in the nucleus (Figure S2).

### 2.2. Prognostic Analysis of LCAT in Tumors

**Figure 3:**
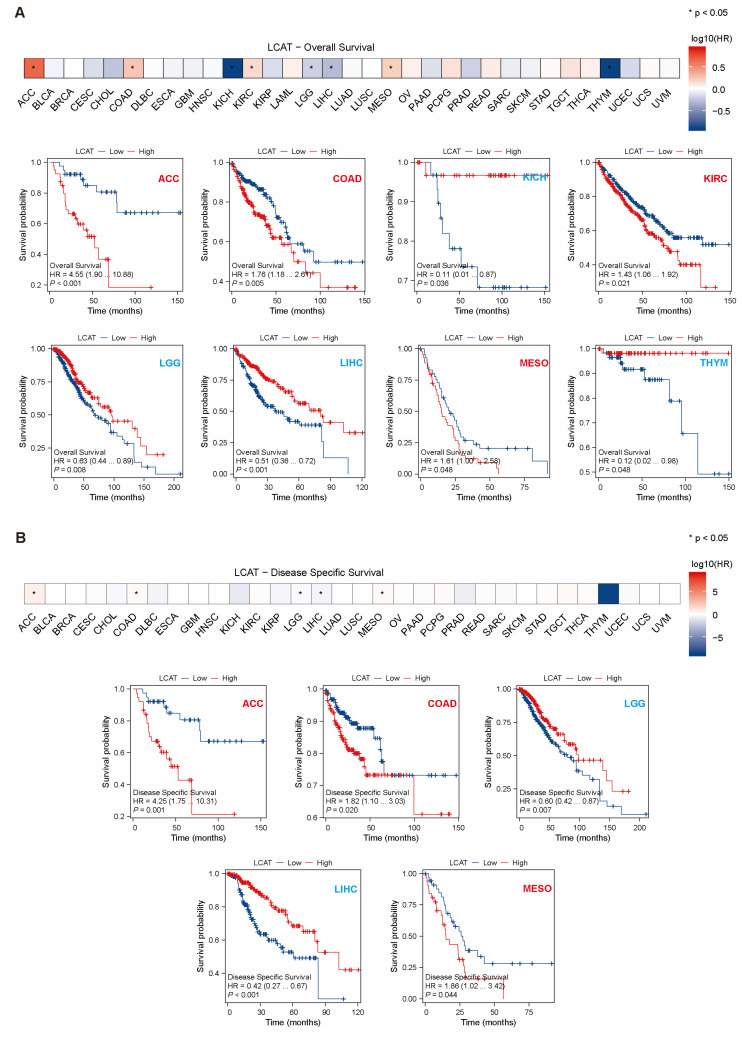
Relationship between *LCAT* expression and overall survival (OS) and disease-specific survival (DSS) in 33 cancer types from the TCGA database. (**A**) Kaplan-Meier survival curves for the relationship between *LCAT* expression and OS in ACC, COAD, KICH, KIRC, LGG, LIHC, MESO, and THYM tumors (*p* < 0.05). (**B**) Kaplan-Meier survival curves for the relationship between *LCAT* expression and DSS in ACC, COAD, LGG, and LIHC tumors (*p* < 0.05). * *p* < 0.05.

**Figure 4:**
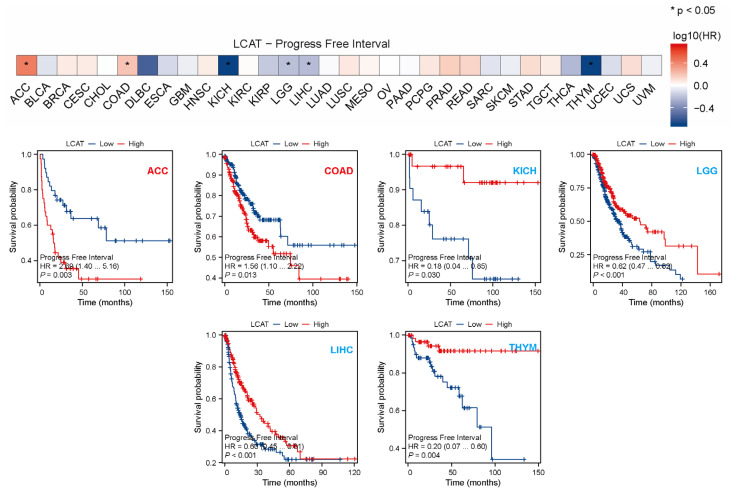
Relationship between *LCAT* expression and overall survival (OS) and progression-free interval (PFI) in 33 cancer types from the TCGA database. Kaplan-Meier survival curves for the relationship between *LCAT* expression and PFI in ACC, COAD, KICH, LGG, LIHC, and THYM tumors (*p* < 0.05). * *p* < 0.05.

To further understand the prognostic value of *LCAT* in different tumors, we analyzed the correlation between *LCAT* expression and OS, DSS, and PFI in tumor patients using univariate Cox regression analysis. The results indicated that in KICH, LGG, LIHC, and Thymoma (THYM) patients, low *LCAT* expression is associated with poor OS prognosis. In ACC, COAD, kidney renal clear cell carcinoma (KIRC), and mesothelioma (MESO) patients, high *LCAT* expression is associated with poor OS prognosis (Figure 3A). Figure 3B analyzed the correlation between *LCAT* expression and DSS in tumor patients, finding that the correlation between *LCAT* expression and DSS in ACC, COAD, LGG, LIHC, and MESO patients is the same as with OS. Figure 4 shows the correlation between *LCAT* expression and PFI in tumor patients; we find that the correlation between *LCAT* expression and PFI in ACC, COAD, KICH, LGG, LIHC, and THYM patients is the same as with OS. The prognostic data of *LCAT* expression and OS, DSS, and PFI in 33 tumor patients are shown in Figure S3.

### 2.3. CNV and SNV Genetic Analysis of LCAT in Tumors

**Figure 5:**
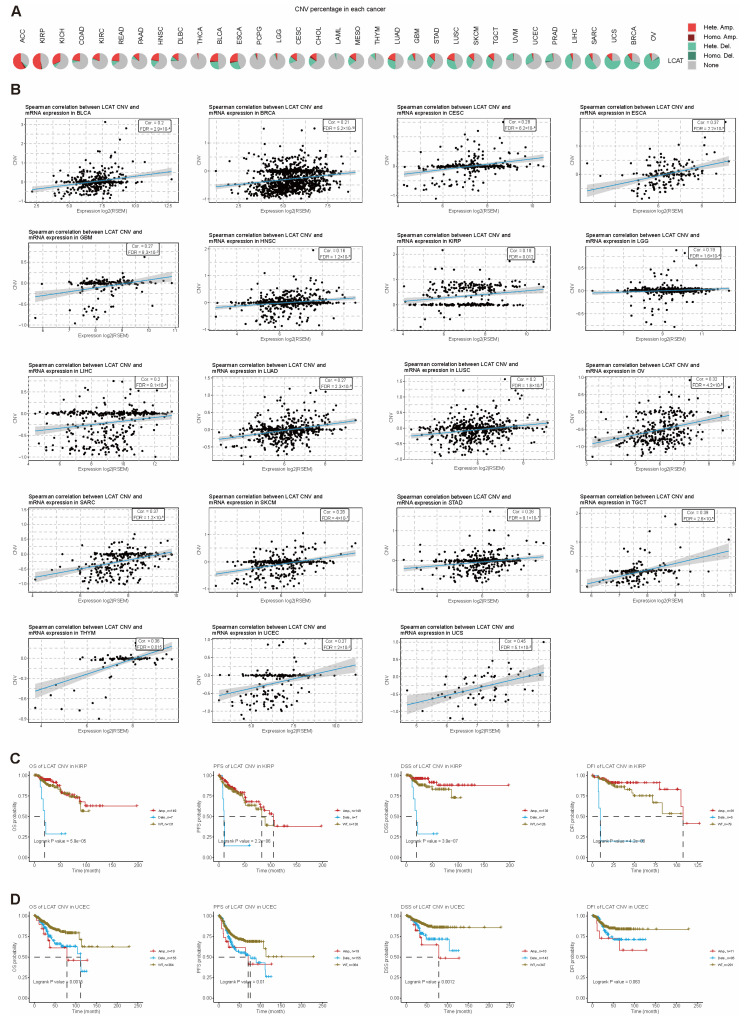
CNV summary and its correlation with *LCAT* expression and survival rates in different types of tumors. (**A**) Pie chart summary of the proportion of different *LCAT* CNVs in 33 cancer types. (**B**) Scatter plot of Spearman correlation between *LCAT* CNV and mRNA expression in various cancer types. (**C**) Survival analysis of *LCAT* CNV in KIRP (OS, PFS, DSS, and DFI). (**D**) Survival analysis of *LCAT* CNV in UCEC (OS, PFS, DSS, and DFI).

CNV is a type of genomic variation where DNA segments exist in different copy numbers within an individual’s genome [15]. CNV can lead to the overexpression or loss of genes, thereby affecting gene function and phenotype [16]. The CNV pie chart shows the composition of the heterozygous/homozygous CNVs of the *LCAT* gene in 33 cancers (Figure 5A). The ACC samples have the highest percentage of samples with total copy number gain and heterozygous gain; the ovarian serous cystadenocarcinoma (OV) samples have the highest percentage of samples with total copy number loss, heterozygous loss, and homozygous loss; the acute myeloid leukemia (LAML) samples show no copy number gain; the THCA samples have the lowest percentage of samples with copy number loss. Additionally, the CHOL samples have the highest percentage of samples with homozygous gain. Our analysis of the correlation between *LCAT* CNV and mRNA expression in 33 tumors found that *LCAT* CNV and mRNA expression are positively correlated in bladder urothelial carcinoma (BLCA), BRCA, cervical squamous cell carcinoma, endocervical adenocarcinoma (CESC), ESCA, GBM, HNSC, KIRP, LGG, LIHC, LUAD, LUSC, OV, SARC, skin cutaneous melanoma (SKCM), STAD, testicular germ cell tumors (TGCTs), THYM, UCEC, and uveal melanoma (UVM) tumors (Figure 5B). In other tumor types, there is no significant correlation between *LCAT* CNV and *LCAT* mRNA expression (Table S1). Further evaluation of the effect of *LCAT* CNV on cancers with the most affected person prognosis showed that *LCAT* CNV is associated with the prognosis of sufferers with COAD, KICH, KIRP, pheochromocytoma and paraganglioma (PCPG), PRAD, UCEC, UVM, CESC, lymphoid neoplasm diffuse large B-cell lymphoma (DLBC), ESCA, LGG, MESO, SARC, THCA, and THYM tumors (Figure S4). Compared to patients with *LCAT* copy number loss, patients with *LCAT* wild-type KIRP have better OS, PFS, DSS, and DFI prognosis (Figure 5C); compared to patients with *LCAT* copy number loss and gain, patients with *LCAT* wild-type UCEC have better OS, PFS, DSS, and DFI prognosis (Figure 5D).

**Figure 6:**
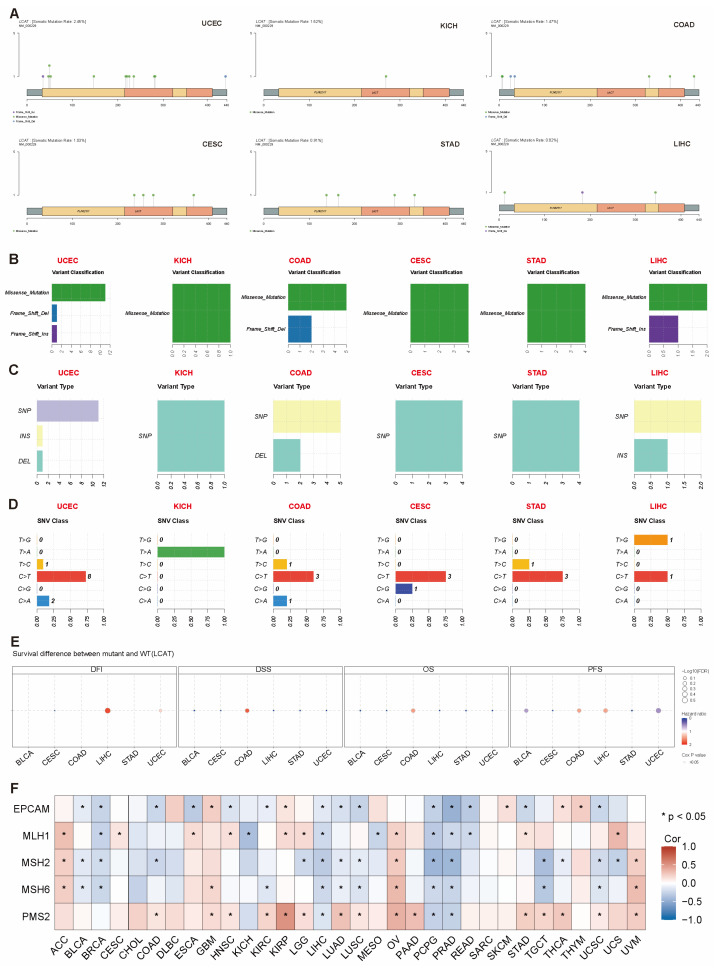
SNV summary and its correlation with *LCAT* expression and survival rates in different types of tumors. (**A**) Lollipop plot showing mutation sites, types, and counts of *LCAT* in selected cancer-type sample sets. (**B**) Number of harmful variations in tumor samples of selected cancer types. (**C**) Count of SNPs and DELs in the input gene set of selected cancer types. (**D**) Count of each SNV category in the input gene set of selected cancer types. (**E**) Bubble chart showing the survival differences between *LCAT* mutants and wild types in different tumor patients. (**F**) Heatmap showing the correlation between *LCAT* expression and MRR genes in different tumor types. * *p* < 0.05.

Single nucleotide variant (SNV) mutations refer to variations where a single nucleotide, the basic unit of DNA, undergoes a change [17]. SNVs can lead to gene mutations and affect patient prognosis. Figure S5 summarizes the expression profiles of SNV mutations in various tumors, with UCSC having the highest proportion of harmful mutations. Figure 6A shows the mutation sites, types, and counts of the *LCAT* gene in UCEC, KICH, COAD, CESC, STAD, and LIHC. Figure 6B summarizes the SNV categories of the *LCAT* gene in the genomes of UCEC, KICH, COAD, CESC, STAD, and LIHC. Further analysis of the relationship between *LCAT* SNV and tumor patient prognosis found that *LCAT* SNV mutations are not significantly correlated with patient prognosis (Figure 6C).

MMR genes play a vital function in DNA repair. When the expression of these genes is affected, it may lead to unrepaired DNA mismatches, causing genomic instability and the accumulation of mutations [18,19]. Therefore, we assessed the correlation between *LCAT* expression and the mutation levels of five MMR genes. The results confirmed that *LCAT* is significantly negatively correlated with MMR genes in BRCA, LIHC, PCPG, and PRAD tumors; *LCAT* is significantly positively correlated with MMR genes in ACC, GBM, OV, and UVM tumors (Figure 6D).

### 2.4. LCAT Methylation Analysis

**Figure 7:**
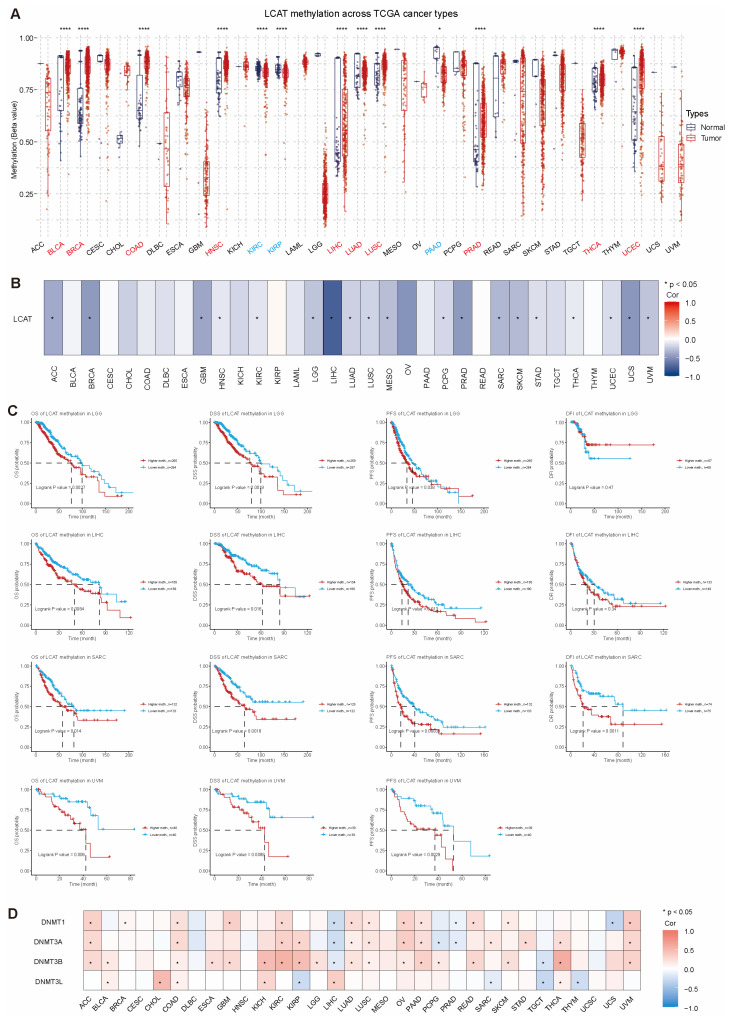
Methylation analysis of *LCAT* expression in different tumor types. (**A**) Methylation differences of *LCAT* between tumor and normal samples in different cancer types. (**B**) Correlation analysis between *LCAT* methylation and mRNA expression in 33 tumors. (**C**) Survival analysis of high and low methylation groups in LGG, LIHC, SARC, and UVM. (**D**) Correlation analysis between *LCAT* expression and DNA methyltransferases in different tumor types. * *p* < 0.05; **** *p* < 0.0001.

Gene methylation refers to the process of adding a CH3 group to the cytosine residue in DNA [20]. This process usually occurs in the promoter regions of genes, and methylation can lead to gene silencing, which can affect cellular function [21,22,23]. Our analysis of *LCAT* methylation level differences in different tumors found that compared to normal tissues, *LCAT* is hypermethylated in BRCA, COAD, HNSC, LUSC, PRAD, and UCEC and hypomethylated in KIRC, KIRP, and pancreatic adenocarcinoma (PAAD). In BLCA, LIHC, LUAD, and THCA, there is no big distinction regarding *LCAT* methylation between normal and tumor tissues (Figure 7A). Except for BLCA, CESC, CHOL, COAD, DLBC, ESCA, KICH, KIRP, LAML, OV, PAAD, rectum adenocarcinoma (READ), TGCT, and THYM, *LCAT* mRNA expression and methylation levels are significantly and drastically correlated in various other tumors (Figure 7B). We analyzed the correlation between *LCAT* methylation tiers and tumor patient prognosis and determined that in STAD, COAD, GBM, LIHC, LGG, KICH, SARC, and UVM, *LCAT* methylation is related to patient prognosis (Figure S6). Compared to the *LCAT* hypomethylation group, the *LCAT* hypermethylation group in LGG, LIHC, SARC, and UVM patients has poorer OS, DSS, and PFS (Figure 7C).

DNA methyltransferases (DNMTs) can add methyl groups to cytosines, leading to chromatin compaction and preventing the binding of transcription factors, thereby silencing related genes [24,25]. In tumors, this silencing may affect the expression of certain genes in tumors, thereby affecting tumor development [26]. We further analyzed the relationship between *LCAT* and four DNMTs. In ACC, BLCA, BRCA, CHOL, COAD, ESCA, GBM, KICH, KIRC, KIRP, LGG, LUAD, LUSC, OV, PAAD, READ, SKCM, STAD, THCA, and UVM tumors, *LCAT* expression is highly positively correlated with the four DNA methyltransferases; in LIHC, PRAD, TGCT, and THYM, *LCAT* expression is highly negatively correlated with the four DNMTs (Figure 7D).

### 2.5. Correlation Analysis of LCAT with m6A Modification

**Figure 8:**
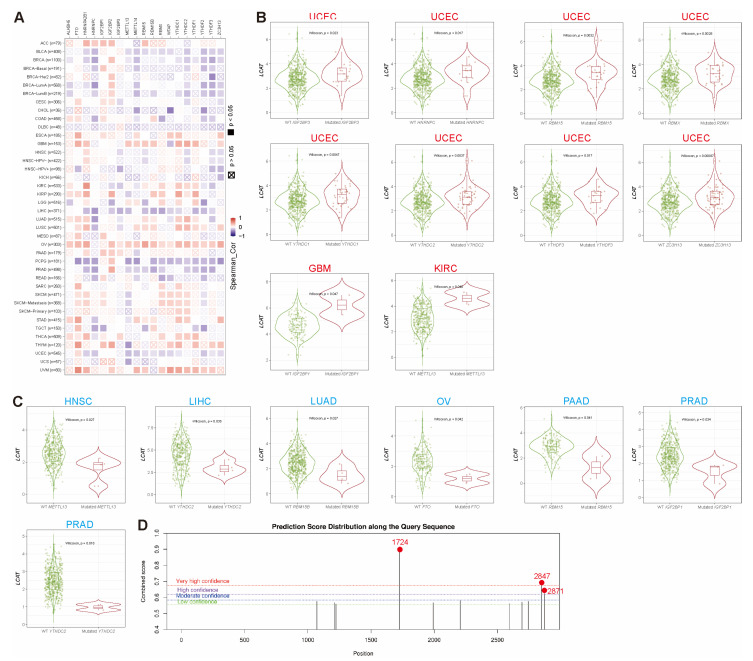
M6A Modification Analysis Related to *LCAT*. (**A**) Heatmap of the correlation analysis between *LCAT* expression and m6A regulatory factors in different tumor types. (**B,C**) Expression of *LCAT* in m6A regulator mutant and wild types in different cancer types (all *p* < 0.05). (**D**) Identification chart of m6A modification sites in the *LCAT* mRNA sequence. * *p* < 0.05.

The most common internal mRNA modification in eukaryotes is m6A, which has become a crucial regulator of gene expression and affects cellular functions like apoptosis, invasion, differentiation, and self-renewal [27,28]. There are three types of m6A regulatory factors: writers, erasers, and readers. While erasers, such as demethylases (like *FTO* and *ALKBH5)*, remove the modification, writers, such as the methyltransferase complex (MTC), catalyze m6A methylation [29]. Reader proteins recognize m6A and determine the fate of target RNAs, playing an essential role in RNA metabolism. The interplay among these modifiers is associated with the onset and progression of cancer [30]. We analyzed the co-expression of *LCAT* and different m6A modification regulators and found that in GBM, LUAD, LUSC, OV, and UVM, *LCAT* expression is significantly positively correlated with the expression of m6A modification regulators; in BLCA, BRCA, LIHC, PCPG, PRAD, and UCSC, *LCAT* and m6A modification regulators are significantly negatively correlated (Figure 8A). The expression of *LCAT* is significantly increased when multiple m6A readers (*IGF2BP3*, *HNRNPC*, *RBMX*, *YTHDC1*, *YTHDC2*, *YTHDF3*, *ZC3H13*) and m6A writers (RBM15) are mutated. Similarly, when m6A readers (*IGF2BP1*) or m6A writers (*METTL13*) are mutated, *LCAT* is significantly highly expressed in GBM and KIRC (Figure 8B). Conversely, in HNSC, LIHC, LUAD, OV, PAAD, and PRAD, the expression of *LCAT* is significantly reduced when certain m6A regulators are mutated (Figure 8C). We also predicted m6A modification sites in the *LCAT* mRNA sequence using the SRAMP web tool. Figure 8D and Table 1 show the m6A modification sites on the *LCAT* gene sequence: sites 1724 (TGGGACCCTGGGATGTTTGGGGACTTTACTATCTAGCACCCCAGT), 2847 (GACCTATCTGTTCCCACCTTGGACTTTGGCAATAAAGGAGCGCCA), and 2871 (TTTGGCAATAAAGGAGCGCCAGACTGGG) have the highest m6A modification scores. The results suggest that m6A regulators can affect tumor progression by regulating the expression of *LCAT.*

### 2.6. LCAT Expression and Immune Correlation Analysis

**Figure 9:**
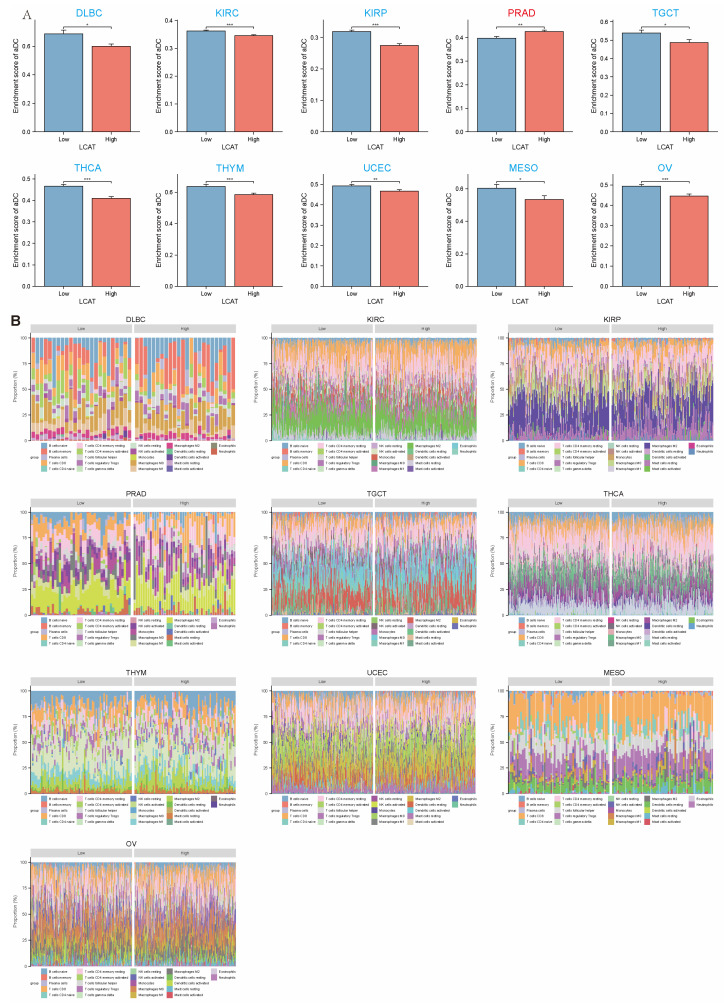
Correlation analysis between *LCAT* expression and immune infiltration in 33 tumor types. (**A**) Bar chart showing the enrichment scores of immune cells in *LCAT* high expression and low expression groups in different types of tumor samples. (**B**) Distribution of multiple immune cell scores in *LCAT* high and low expression groups in selected tumors. * *p* < 0.05; ** *p* < 0.01; *** *p* < 0.001.

As shown in Figure 9A, compared to the *LCAT* low expression group, the *LCAT* high expression group has lower immune cell enrichment scores in DLBC, KIRC, KIRP, TGCT, THCA, THYM, UCEC, MESO, and OV tumor samples. Conversely, in PRAD, the *LCAT* high expression group has higher immune cell enrichment scores. In the remaining tumors, there is no difference in immune cell enrichment scores between the *LCAT* low expression group and the high expression group (Figure S7). The distribution of multiple immune cell scores in the *LCAT* high and low expression groups in DLBC, KIRC, KIRP, TGCT, THCA, THYM, UCEC, MESO, OV, and PRAD is shown in Figure 9B.

**Figure 10:**
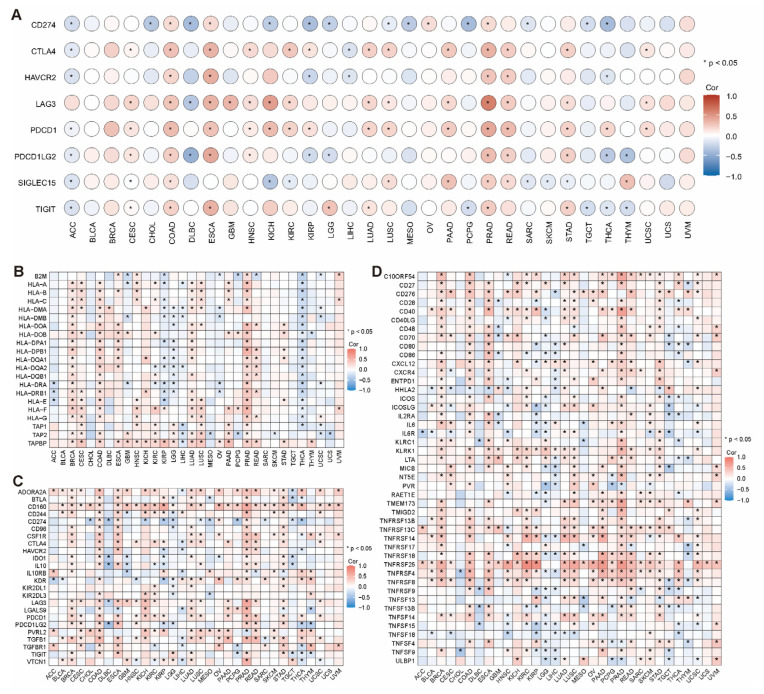
Correlation analysis of *LCAT* expression with immune-related genes across 33 tumor types. Correlation analysis of *LCAT* expression with immune checkpoints (**A**), MHC molecules (**B**), immune suppressors (**C**) and immune stimulatory factors (**D**) in 33 tumor types. * *p* < 0.05.

Immune checkpoint molecules play a key role in regulating the activity of the immune system, especially in preventing excessive autoimmune responses [31]. However, in the tumor microenvironment, the abnormal expression of these molecules may suppress anti-tumor immune responses, thereby promoting tumor growth and development [32,33]. We analyzed the correlation between *LCAT* expression and immune checkpoints in multiple tumors using the TIMER 2.0 database. The results showed that in ACC, *LCAT* expression is significantly negatively correlated with multiple immune checkpoint molecules; in CESC, COAD, ESCA, PRAD, READ, and STAD, *LCAT* expression is significantly positively correlated with multiple immune checkpoint molecules (Figure 10A). The MHC, also known as the human leukocyte antigen (HLA) system, is responsible for presenting antigens to T lymphocytes, initiating a specific immune response [34,35]. Some tumor cells can evade recognition and attack by the immune system by downregulating MHC expression or altering its structure [36,37,38]. The heatmap in Figure 10B shows the correlation between *LCAT* expression and MHC molecules. In BRCA, COAD, LUAD, and PRAD, *LCAT* is significantly positively correlated with almost all MHC molecules. In THCA, *LCAT* is significantly negatively correlated with almost all immune stimulatory factors. Furthermore, we analyzed the relationship between *LCAT* expression and immune suppressors and determined that *LCAT* expression is positively correlated with the expression of immune suppressors in various tumors. *LCAT* showed a substantial and positive correlation with the expression of nearly all immunosuppressive factors in BRCA, COAD, ESCA, LUAD, LUSC, PRAD, READ, and STAD (Figure 10C).

Immune stimulatory factors are a class of substances that can activate and enhance the body’s immune response. These factors include cytokines, chemokines, and other signaling molecules. They promote the activation and function of immune cells by binding to specific receptors, thereby improving the body’s ability to recognize and clear tumor cells [39]. As shown in Figure 10D, in BRCA, COAD, ESCA, KIRC, LUAD, LUSC, PRAD, READ, SKCM, STAD, and UCSC, *LCAT* expression is positively correlated with almost all immune stimulatory factors.

### 2.7. TMB and MSI Analysis Related to LCAT

**Figure 11:**
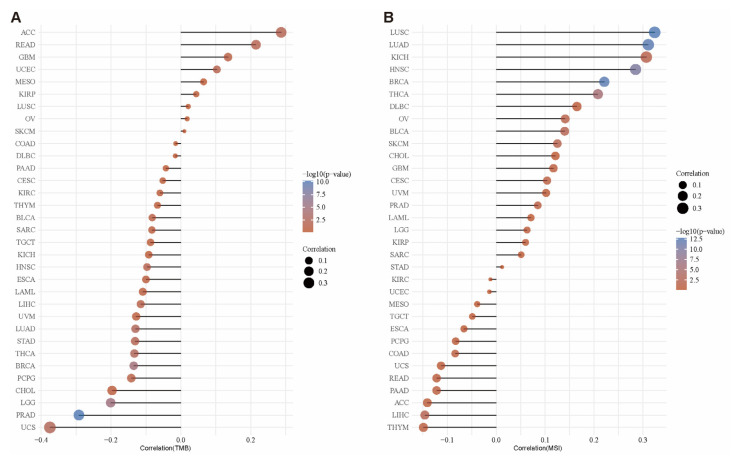
Correlation between *LCAT* expression and TMB and MSI. (**A**) Lollipop plot of the correlation between *LCAT* expression and TMB in pan-cancer. (**B**) Lollipop plot of the correlation between *LCAT* expression and MSI in pan-cancer.

The quantity of somatic mutations present in tumor cells is known as the tumor mutation burden (TMB), and it is typically represented as the number of mutations per megabase [40]. TMB, as a biomarker, has potential value in predicting the response of certain cancer patients to immunotherapy [41,42]. In KICH, HNSC, ESCA, LAML, LIHC, UVM, LUAD, STAD, THCA, BRCA, PCPG, CHOL, LGG, PRAD, and uterine carcinosarcoma (UCS), *LCAT* expression is negatively correlated with TMB. In ACC, READ, GBM, and UCEC, *LCAT* expression and TMB are positively correlated (Figure 11A).

Microsatellite instability (MSI) is a form of genomic instability that typically occurs in cells with defective DNA repair mechanisms [43]. In UCS, READ, PAAD, ACC, LIHC, and THYM, *LCAT* expression and MSI are negatively correlated. In LUSC, LUAD, KICH, HNSC, BRCA, THCA, DLBC, OV, BLCA, SKCM, CHOL, GBM, CESC, and UVM, *LCAT* expression and MSI are positively correlated (Figure 11B).

### 2.8. Correlation Analysis of LCAT Expression and Drug Sensitivity

**Figure 12:**
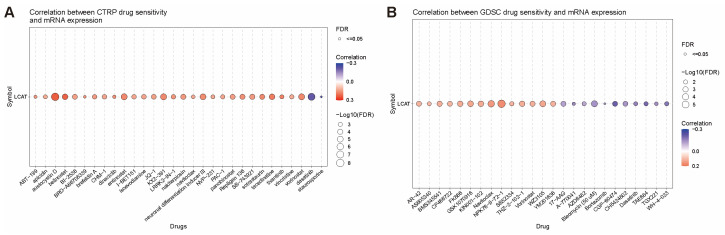
Correlation Analysis of *LCAT* expression and drug sensitivity. (**A**) Correlation analysis of *LCAT* expression with CTRP drug sensitivity (top 30). (**B**) Correlation analysis of *LCAT* gene expression with GDSC drug sensitivity.

We used the “GDSC” and “CTRP” modules of the GSCA online tool to analyze the correlation between *LCAT* expression and the IC50 of various anti-cancer drugs. As shown in the bubble chart in Figure 12A, in GDSC, the IC50 of almost all anti-cancer drugs is significantly positively correlated with *LCAT* mRNA expression. Among them, the positive correlation between *LCAT* expression and the IC50 of BRD-K01737880 is the strongest. The negative correlation between *LCAT* expression and the IC50 of BRD-staurosporine is the strongest. In the CTRP database, *LCAT* mRNA expression is significantly correlated with the IC50 of various anti-cancer drugs. The positive correlation between *LCAT* expression and the IC50 of NPK76-II-72-1 is the strongest. The negative correlation between *LCAT* expression and the IC50 of CGP-60474 is the strongest (Figure 12B).

### 2.9. Molecular Mechanisms by Which LCAT Affects Tumor Progression

Based on the results of Figure 3 and Figure 4, we can conclude that *LCAT* expression significantly affects the progression of ACC, COAD, LGG, and LIHC tumors. To further understand how *LCAT* promotes the progression of ACC and COAD tumors and how it inhibits the progression of LGG and LIHC tumors, we divided ACC, COAD, LGG, and LIHC into two groups based on *LCAT* expression and performed functional enrichment analysis on the differential genes between the two groups.

The GO enrichment analysis shows that in ACC, these differential genes are mainly involved in DNA replication, fibrillar collagen trimer, and CXCR chemokine receptor binding (Figure S8A–C). In COAD, these differential genes are mainly involved in humoral immune response mediated by circulating immunoglobulin, immunoglobulin complex, and antigen binding (Figure S8D–F). In addition, the signaling pathways involved via these differential genes in ACC and COAD are consistent. In LGG, the differential genes between the high and low *LCAT* expression groups are mainly involved in humoral immune response mediated by circulating immunoglobulin, immunoglobulin complex, and immunoglobulin receptor binding (Figure S8G,I). In LIHC, the differential genes between the high and low *LCAT* expression groups are mainly involved in the carboxylic acid catabolic process, HDL particles, and oxidoreductase activity (Figure S8J–L).

**Figure 13:**
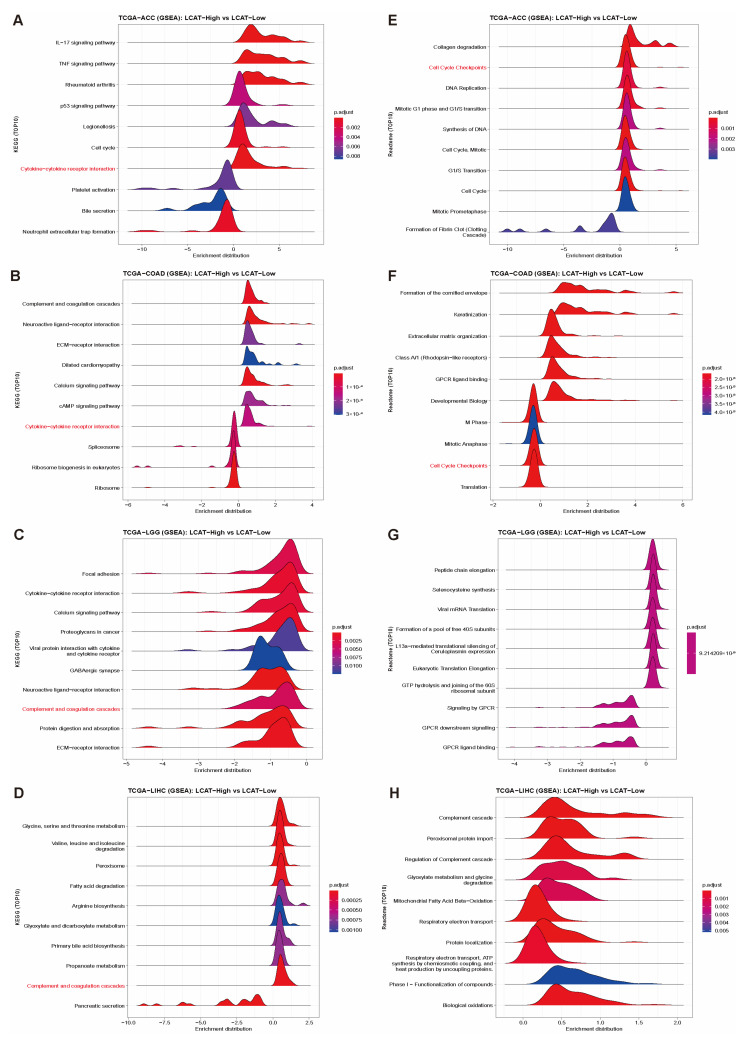
Functional enrichment analysis of *LCAT* affecting the progression of ACC, COAD, LGG, and LIHC tumors. Mountain plots showing GSEA analysis of *LCAT* high and low expression groups in ACC (**A**), COAD (**B**), LGG (**C**), and LIHC (**D**) tumors. Mountain plots showing Reactome analysis of *LCAT* high and low expression groups in ACC (**E**), COAD (**F**), LGG (**G**), and LIHC (**H**) tumors.

The KEGG enrichment analysis results indicate that *LCAT* is most likely to promote ACC tumor progression through the IL-17 signaling pathway (Figure 13A); *LCAT* is most likely to promote COAD tumor progression through complement and coagulation cascades (Figure 13B). In addition, in ACC and COAD tumors, *LCAT* is likely to promote tumor progression through cytokine–cytokine receptor interaction (Figure 13A,B). *LCAT* is most likely to inhibit LGG tumor progression by affecting focal adhesion formation (Figure 13C); *LCAT* is most likely to inhibit LIHC tumor progression through glycine, serine, and threonine metabolism (Figure 13D). In LGG and LIHC tumors, *LCAT* is likely to inhibit tumor progression through complement and coagulation cascades (Figure 13C,D).

The Reactome enrichment analysis results show that *LCAT* is most likely to promote ACC tumor progression through collagen degradation (Figure 13E); *LCAT* is most likely to promote COAD tumor progression by affecting cornified envelope formation (Figure 13F). In addition, in ACC and COAD tumors, *LCAT* is likely to promote tumor progression by regulating cell cycle checkpoints (Figure 13E,F). *LCAT* is most likely to inhibit LGG tumor progression by affecting peptide chain elongation (Figure 13G); *LCAT* is most likely to inhibit LIHC tumor progression through complement cascades (Figure 13H).

## 3. Discussion

Elevated cholesterol levels are considered a prerequisite for cancer cell proliferation and tumor progression [44]. Mitochondrial cholesterol levels can induce resistance to apoptotic signals, and cholesterol also regulates the physicochemical properties of the cell membrane, including lipid rafts and signaling receptors such as the Epidermal Growth Factor Receptor [2]. The role of *LCAT* in the maturation of HDL and the conversion of free cholesterol into cholesterol ester may affect the cholesterol content and homeostasis in cancer cells [45]. Therefore, there is a growing recognition of the multifaceted roles of metabolic enzymes such as *LCAT* in tumorigenesis and immune regulation. Our comprehensive analysis of the role of *LCAT* in various cancers reveals its potential as a biomarker and therapeutic target, emphasizing the necessity of understanding its mechanisms of action.

The role of *LCAT* in cancer biology is complex and context-dependent, reflecting its dual nature as both a tumor suppressor and a potential promoter of tumor progression. This study provides a comprehensive analysis of the expression, epigenetic regulation, immune interactions, and therapeutic implications of *LCAT* across multiple cancer types. Our findings highlight the importance of understanding the multifaceted roles of *LCAT* in tumor biology, particularly its involvement in lipid metabolism, immune modulation, and epigenetic regulation. The dual nature of *LCAT* is evident in its contrasting roles across different cancer types. In low-grade glioma (LGG) and liver hepatocellular carcinoma (LIHC), low *LCAT* expression is associated with poor prognosis, suggesting a tumor-suppressive function. This aligns with previous studies showing that *LCAT* inhibits tumor progression by modulating cholesterol metabolism and enhancing HDL functionality, which may suppress tumor growth and immune evasion [4,5]. Conversely, in adrenocortical carcinoma (ACC) and colon adenocarcinoma (COAD), high *LCAT* expression correlates with worse outcomes, indicating a potential oncogenic role. This duality underscores the importance of context-specific mechanisms, where *LCAT* may either promote or inhibit tumor progression depending on the tumor microenvironment and genetic background. For instance, in LGG and LIHC, *LCAT* likely exerts its tumor-suppressive effects by regulating lipid metabolism and immune responses. Our functional enrichment analysis revealed that *LCAT* inhibits tumor progression in these cancers through pathways such as complement and coagulation cascades and oxidoreductase activity, which are critical for maintaining cellular homeostasis and suppressing tumorigenesis. In contrast, in ACC and COAD, *LCAT* may promote tumor progression by enhancing DNA replication and cytokine–cytokine receptor interactions, which are essential for tumor cell proliferation and survival. These findings suggest that the role of *LCAT* in cancer is not uniform but rather depends on the specific molecular and cellular context of each tumor type.

The expression of *LCAT* in tumor cell lines and the website-based predictions of its subcellular localization both indicate significant nuclear expression of *LCAT*. Previous studies have also shown that gene expression in the nucleus is closely related to tumor progression. The aberrant expression of genes in the nucleus can lead to the overproduction of key oncoproteins or the loss of tumor suppressor proteins, thereby affecting cell cycle control, DNA repair, apoptosis, and other processes, promoting the occurrence and development of tumors [46].

In terms of genomic alterations, our investigation into the relationship between CNVs and SNVs with *LCAT* expression reveals more layers of genetic complexity affecting cancer progression. In various tumors, *LCAT*s CNV and mRNA expression show a positive correlation, impacting the survival of patients with multiple tumors, especially KIRP and USEC. The correlation between CNVs and *LCAT* expression underscores the necessity of understanding how genomic alterations lead to the dysregulation of metabolic enzymes in the tumor environment. Notably, there is a lack of significant association between SNV mutations in *LCAT* and survival outcomes. Our study results suggest that *LCAT* expression is influenced by DNA methylation patterns. In various cancer types, hypermethylation is associated with reduced expression, and *LCAT* methylation levels significantly impact tumor progression in LGG, LIHC, SARC, and UVM. These insights suggest that future research should investigate whether demethylating drugs can enhance *LCAT* expression, potentially restoring its tumor-suppressing function.

N6-methyladenosine (m6A) modification affects the progression of various cancers by regulating the expression of tumor-associated genes [27,47,48,49,50]. For instance, in bladder cancer, the upregulation of METTL3 enhances the methylation of CDCP1 mRNA, promoting its translation and tumor progression [51]. In colorectal cancer, METTL3 promotes the stability of SOX2 mRNA by catalyzing its m6A modification, thereby promoting tumor development [52]. The dual role of m6A in cancer further highlights its ability to promote or inhibit tumorigenesis, depending on the context. The interaction between *LCAT* and m6A regulatory factors provides a new avenue for understanding post-transcriptional regulatory mechanisms in cancer. Identifying m6A modification sites on *LCAT* mRNA may help develop targeted therapies that alter RNA structure, thereby enhancing the anti-tumor effects of existing therapies. Our analysis indicates that in GBM, LUAD, LUSC, OV, and UVM, *LCAT* expression is significantly positively correlated with the expression of m6A modification factors; in BLCA, BRCA, LIHC, PCPG, PRAD, and UVM, *LCAT* expression is significantly negatively correlated with the expression of m6A modification factors. *LCAT* expression is significantly elevated when multiple m6A readers and writers are mutated. The distribution of *LCAT* expression is related to m6A modification sites.

Immune cells in the tumor microenvironment can regulate the behavior of tumor cells by secreting cytokines and metabolic products, including promoting tumor angiogenesis, invasion, and metastasis [53]. An in-depth study of these complex interactions is crucial for developing new immunotherapeutic strategies and predicting tumor treatment responses. Our immunological analysis suggests that high *LCAT* expression is associated with a reduction in immune cell infiltration in several cancers, indicating that *LCAT* may contribute to immune evasion mechanisms. As depicted in Figure 10, there is a significant correlation between *LCAT* expression and various immune regulatory factors. Furthermore, we analyzed the correlation between *LCAT* expression and TMB as well as MSI in different tumors and found that *LCAT* expression is significantly associated with the levels of TMB and MSI across various cancers. These correlations are crucial, suggesting that *LCAT* may influence anti-tumor immunity by modulating immune regulatory factors. Therefore, targeting *LCAT* could potentially alter lipid metabolism and enhance the efficacy of immune checkpoint blockade in drug-resistant tumors.

Our analysis of the correlation of *LCAT* with drug sensitivity revealed its potential as a predictive biomarker for chemotherapy response. In the GDSC and CTRP databases, *LCAT* expression was significantly correlated with the IC50 of various chemotherapeutic drugs, suggesting that *LCAT* may influence drug resistance or sensitivity in cancer cells. For example, high *LCAT* expression was associated with increased resistance to BRD-K01737880 and NPK76-II-72-1, while low *LCAT* expression correlated with sensitivity to BRD-staurosporine and CGP-60474. These findings suggest that *LCAT* could be used to stratify patients for personalized therapy, particularly in cancers where it modulates drug sensitivity.

In ACC and COAD, *LCAT* expression levels significantly affect tumor progression. The GO enrichment analysis of the *LCAT* high and low expression groups in ACC and COAD suggests that *LCAT* may promote tumor progression in ACC and COAD by affecting DNA replication and immune responses. The KEGG enrichment analysis further supports this view, showing that *LCAT* may promote ACC tumor progression through the IL-17 signaling pathway and promote COAD tumor progression through complement and coagulation cascades. Additionally, the interaction between cytokines and cytokine receptors may be regulated by *LCAT* in both cancers, explaining the common mechanism of action of *LCAT* in different cancers. Unlike ACC and COAD, *LCAT* may play a role in inhibiting tumor progression in LGG and LIHC. The GO enrichment analysis shows that in LGG, *LCAT*-related differential genes are mainly involved in humoral immune responses and immunoglobulin receptor binding, whereas in LIHC, they involve carboxylic acid metabolism, HDL particles, and oxidoreductase activity. These results suggest that *LCAT* may inhibit the progression of these two cancers by regulating immune responses and metabolic processes. Complement and coagulation cascades may be regulated by *LCAT* in LGG and LIHC, explaining the common mechanism of action of *LCAT* in inhibiting tumor progression. The Reactome enrichment analysis further emphasizes the role of *LCAT* in regulating peptide chain elongation and complement cascades, which may be key molecular mechanisms for its inhibitory effect on LGG and LIHC tumor progression.

Although our study provides valuable insights into the role of *LCAT* in cancer, the mechanism by which *LCAT* has a dual nature in different cancer types requires further investigation. Future studies should also explore the interactions between *LCAT* and other metabolic enzymes in the tumor microenvironment, as well as the role of *LCAT* in regulating immune cell function and immunotherapy response.

## 4. Materials and Methods

### 4.1. LCAT Expression Profile Data Analysis

The Human Protein Atlas (HPA) website furnished us with records on *LCAT* expression in healthful tissues. The UALCAN (https://ualcan.path.uab.edu/) database provided us with records on *LCAT* expression in tumor tissues (accessed on 11 October 2024). From the TCGA database, we extracted medical records and raw RNAseq data from tumor and normal tissues, which were obtained from the Xiantao Academic website (https://www.xiantaozi.com/) (accessed on 5 October 2024). By changing counts to Transcripts Per Million (TPM) and the usage of log2 (TPM+1) transformation, the uncooked records were normalized. We examined the variations in *LCAT* expression between the tumor and adjoining regular tissues. We examined the expression of *LCAT* in quite a number of pathological ranges of tumors for sufferers with medical records in order to reap a higher appreciation of *LCAT* expression in tumors. The expression level of *LCAT* in tumors was divided into high and low expression groups based on the median value of *LCAT* expression as the cut-off point. The Wilcoxon signed-rank check was used to consider the statistical differences.

### 4.2. Human Protein Atlas (HPA)

HPA provided the expression data for LCAT in tumor cell lines and tumor tissues (https://www.proteinatlas.org) (accessed on 18 October 2024) [54]. LCAT subcellular localization information was found using the“CELL ATLAS” module of HPA.

### 4.3. Survival Analysis

Survival evaluation was performed using tumor-affected person RNA-seq records from the TCGA database, obtained via XianTao Academic (retaining samples with scientific information). Patients with a range of tumor types were analyzed for OS, DSS, and PFI by using univariate Cox regression analysis. The R software program (3.6.3) was used to analyze the data. The R package deal “survival (3.2-10)” was used once for the statistical evaluation of the survival data, and the R bundle “survminer (0.4.9)” was used for visualization.

### 4.4. CNV Mutation Analysis

We input “*LCAT*” into the search template of the Gene Set Cancer Analysis (GSCA) (https://guolab.wchscu.cn/GSCA/ (accessed on 25 October 2024)) [55] online tool. All tumor types were selected, and the CNV summary, CNV & Expression, and CNV & Survival modules were checked before initiating the search. The GSCA online tool downloaded CNV data from 11,495 samples and processed them using GISTIC2.0. Pie charts illustrate the percentage distribution of different types of CNV mutations in 33 different tumor types (total copy number gain, total copy number loss, heterozygous gain, heterozygous loss, homozygous gain, and homozygous loss). The percentage of heterozygous gain is shown in red, the percentage of heterozygous loss in brown, the percentage of homozygous gain in light green, the percentage of homozygous loss in dark green, and the percentage of no gene CNV mutation in gray. The Spearman correlation between *LCAT* CNV and mRNA expression in 33 tumors is shown in scatter plots with FDR-adjusted *p*-values. The survival times and statuses within the wild–type (WT), copy number gain, and copy number loss groups of the samples were modeled using the R software package “survival.” To evaluate variations in the groups’ survival rates, logrank tests were used, as generated and analyzed by the GSCA website. The OS, PFS, DSS, and PFI survival characteristics of *LCAT* CNV in KIRP and UCEC are shown using survival curves.

### 4.5. SNV Mutation Analysis

SNV information from 10,234 samples for 33 different cancer types was gathered from the TCGA database via the GSCA web tool. Missense mutation, nonsense mutation, frame shift insertion, splice site mutation, frame shift deletion, in-frame deletion, and in-frame insertion are the seven types of mutations that we examined. The amount of detrimental *LCAT* variants in the chosen cancer types is known as the variant classification. Variant type: the quantity of SNPs and DELs found in the chosen cancer kinds’ *LCAT* concentrations. SNV categories: the number of each SNV category found in the chosen cancer types that are concentrated in *LCAT*. The survival differences between wild-type and mutant *LCAT* in 33 cancer types were summarized using bubble charts.

### 4.6. MMR Mutation Analysis

Spearman correlation data between *LCAT* expression and five MMR genes were assessed using TIMER 2.0 (http://compbio.cn/timer2/) (accessed on 21 October 2024) [56], with the results visualized using R package “ggplot2 (version 3.3.3)”. http://timer.cistrome.org/.

### 4.7. Methylation Analysis

The TCGA database was used to retrieve the Illumina methylation and mRNA expression data used in the GSCA online tool. Various methylation levels resulted from the acquisition of multiple methylation sites within a gene region. The relationship between gene mRNA expression and methylation levels was examined using Spearman correlation analysis. Clinical data on tumor samples from 33 extraordinary cancer types was acquired once by way of GSCA from TCGA and posted research. Clinical survivl and methylation data were combined based on sample size. The tumor samples were divided into excessive and low methylation groups based totally on the median methylation level. The “survival” R software program package deal was used once to suit the survival instances and statuses of the two groups, as generated and analyzed by the GSCA website. A Cox proportional-hazards mannequin was used to decide the hazard ratio between the excessive and low methylation groups, and logrank was employed to see whether or not the editions in survival quotes between the two agencies were statistically significant. Spearman correlation between *LCAT* mRNA expression and methyltransferase genes (*DNMT1*, *DNMT3A*, *DNMT3B*, and *DNMT3L*) in 33 tumors was retrieved from the TIMER 2.0 database.

### 4.8. M6A Modification Analysis

Spearman correlation analysis between *LCAT* mRNA expression levels and 19 m6A regulatory factors in different cancers was conducted using the “Exploration-Gene_Corr” module in TIMER 2.0. The “Prediction” module of the sequence-based RNA adenosine methylation site predictor (SRAMP) web tool (http://www.cuilab.cn/sramp/ (accessed on 23 October 2024)) [57] was used to predict the m6A modification sites in *LCAT*. The specific operation is as follows: (1) Input the FASTA *LCAT* mRNA sequence in Mature mRNA mode; (2) analyze RNA secondary structure—NO; (3) tissue selection is universal; (4) show query sequence as RNA; (5) finally, click “submit”.

### 4.9. Immune-Related Analysis

Data on *LCAT* expression and immune cell expression in different tumor types were obtained from the TCGA database through XianTao Academic. The immune cell expression data included the infiltration levels of various immune cell types, such as naive B cells, memory B cells, plasma cells, CD8+ T cells, naive CD4+ T cells, memory resting CD4+ T cells, memory-activated CD4+ T cells, follicular helper T cells, regulatory T cells (Tregs), gamma delta T cells, resting NK cells, activated NK cells, monocytes, M0 macrophages, M1 macrophages, M2 macrophages, resting dendritic cells, activated dendritic cells, resting mast cells, activated mast cells, eosinophils, and neutrophils. The ssGSEA algorithm provided by the R package GSVA [version 1.46.0] was used to calculate the immune infiltration levels corresponding to these immune cell types based on the transcriptomic data. The ggplot2 package deal was used once to visualize the statistics; the appropriate statistical techniques were utilized for statistical evaluation (statistics bundle and auto package). Spearman correlation data between *LCAT* expression and immune-related genes (immune checkpoints, immune stimulatory factors, immune inhibitors, and major histocompatibility complex (MHC) molecules) were downloaded from the TIMER 2.0 online website. The results were visualized using the R software package “ggplot2 (version 3.3.3).” Finally, correlation data between *LCAT* mRNA expression and MSI and TMB expression were obtained from the ASSISTANT for ClinicaL Bioinformatics website (https://www.aclbi.com/static/index.html (accessed on 28 October 2024), with correlation analysis performed using Spearman analysis.

### 4.10. Drug Sensitivity Analysis

We used the online tool GSCA to analyze the correlation between *LCAT* mRNA expression and drug sensitivity in multiple tumors. The GSCA online tool collected the IC50 of various small molecule drugs in cell lines from the Genomics of Drug Sensitivity in Cancer (GDSC) and Genomics of Therapeutics Response Portal (CTRP) databases, along with corresponding mRNA gene expression. The mRNA expression facts and drug sensitivity information were merged using the GSCA online tool. The correlation between gene mRNA expression and drug IC50 was analyzed once using Pearson correlation. The *p*-values were adjusted via FDR. We examined the relationship between *LCAT* mRNA expression and treatment sensitivity in a number of malignancies using the web application GSCA. The GDSC and CTRP databases provided the GSCA online tool with the IC50 of several small molecule medicines in the cell lines, as well as the related mRNA gene expression. The GSCA online tool combined the data on medication sensitivity and mRNA expression. Pearson correlation was used to examine the relationship between medication IC50 and gene mRNA expression. FDR was used to alter the *p*-values.

### 4.11. Functional Enrichment Analysis

Comprehensive Analysis on Multi-Omics of Immunotherapy in Pan-cancer (CAMOIP) (https://www.camoip.net/) (accessed on 3 November 2024) [58] is a comprehensive analysis tool that is specifically designed for processing and analyzing expression and mutation data in TCGA and immune checkpoint inhibitor treatment projects. This study used the “Pathway Enrichment Analysis” module of CAMOIP; we clicked on “GSEA,” selected “TCGA-Cohort” in Step 1, and subsequently input the tumor and gene of interest. In Step 2, KEGG, GO-BP, GO-CC, and GO-MF were chosen for analysis.

## 5. Conclusions

This study unveils the multifaceted roles of *LCAT* in cancer, particularly its key involvement in tumor immune modulation and progression. The expression levels of *LCAT* are closely associated with the prognosis of patients across various cancers, potentially serving as a biomarker for predicting treatment response. The impact of genetic and epigenetic variations on *LCAT* function offers new insights for cancer therapy. The role of *LCAT* in regulating immune responses in the tumor microenvironment and drug sensitivity underscores its potential in cancer treatment. These findings provide a scientific basis for developing personalized therapeutic strategies targeting *LCAT*, highlighting its significant role in oncology.

## Figures and Tables

**Table 1:** Prediction of m6A modification sites in mature mRNA of *LCAT*.

Position	Sequence Context	Score
1072	CGCAGATGCTGCGGCAGATGAGA CTGACCAAGACTGAGCGGGAGC	0.704
1212	ATCCAGATGACGTGGACCAGGG ACAAGTACATGACTGAGACCTGG	0.603
1223	GTGGACCAGGGACAAGTACATG ACTGAGACCTGGGACCCCAGCCA	0.582
1724	TGGGACCCTGGGATGTTTGGGG ACTTTACTATCTAGCACCCCAGT	0.903
1991	GAGACAGCTGAGCTGAGGCCTG ACTTTTTCAATAAAACATTGTGT	0.584
2205	CCCACTCCCACACCAGATAAGG ACAGCCCAGTGCCGCTTTCTCTG	0.579
2593	TCCCTTCTCCCACCACACTGTGA CTCTCAGTTGTCTAACCCAGGG	0.559
2694	TGGTCAGTCACAGCCACACCAGA CTCTGGGCCAAGCCCCACCACT	0.61
2743	CCTTGGCCCCCACCCACCAAGGA CAAGATGCCCAGCCCAGGATCG	0.641
2847	GACCTATCTGTTCCCACCTTGGA CTTTGGCAATAAAGGAGCGCCA	0.76
2871	TTTGGCAATAAAGGAGCG CCAGACTGGG-----------------	0.563

## Data Availability

Raw data1-2 can be downloaded at https://pan.quark.cn/s/55bb0239bb45 (accessed on 3 February 2025). Raw data3-12 can be downloaded at https://pan.quark.cn/s/884b9fb0313f (accessed on 3 February 2025).

## Supplementary Materials

Supplementary material-figure can be downloaded at https://pan.quark.cn/s/4ce2fde984c8 (accessed on 3 February 2025). supplementary material-table1 can be downloaded at https://pan.quark.cn/s/018517336c51 (accessed on 3 February 2025).
